# Statistical factorial designs for optimum production of thermostable α-amylase by the degradative bacterium *Parageobacillus thermoglucosidasius* Pharon1 isolated from Sinai, Egypt

**DOI:** 10.1186/s43141-021-00123-4

**Published:** 2021-02-01

**Authors:** Ali M. Saeed, Einas H. El-Shatoury, Hayam A. E. Sayed

**Affiliations:** Department of Microbiology, Faculty of Science, Ain Shams University, Cairo, Egypt

**Keywords:** Thermostable amylase, Thermophilic, *Parageobacillus thermoglucosidasius*, Hot spring, Full factorial design, Response surface optimization

## Abstract

**Background:**

This study aimed to isolate potent thermophilic and amylolytic bacteria from a hot spring of Pharaoh’s bath, Sinai, Egypt, and screen its degradative activity. The amylolytic activity was further optimized using a statistical full factorial design followed by response surface methodology.

**Results:**

A thermophilic bacterium was isolated from the hot spring of Pharaoh’s Bath, Sinai, Egypt. The isolate produced amylase, cellulase, and caseinase and was identified by 16S rRNA gene sequencing as *Parageobacillus thermoglucosidasius* Pharon1 (MG965879). A growth medium containing 1% soluble starch was found to optimize the amylase production. Dinitrosalycalic acid method (DNS) was used to estimate the amount of reducing sugar produced. Statistical full factorial and response surface designs were employed to optimize physical variables affecting the α-amylase production and determine the significant interactions of the studied variables during the fermentation process. According to the results obtained by the response optimizer, the maximum amylase activity reached 76.07 U/mL/ min at 54.1°C, pH 5.6 after 98.5 h incubation under aerobic conditions. Moreover, the produced enzyme was thermostable and retained most of its activity when exposed to a high temperature of 100°C for 120 min. Maximum enzyme activity was attained when the enzyme was incubated at 70°C for 30 min.

**Conclusions:**

This is the first report of the production of thermostable α-amylase by the potent thermophilic *Parageobacillus thermoglucosidasius.* The enzyme endured extreme conditions of temperature and pH which are important criteria for commercial and industrial applications.

**Supplementary Information:**

The online version contains supplementary material available at 10.1186/s43141-021-00123-4.

## Background

Amylases are important enzymes that play a pivotal role in biotechnology. They are produced by plants, animals, and microorganisms. Amylases are widely used in baking and bread industry, fermentations, textiles, alcohols, pharmaceuticals, and detergents [1]. Moreover, they are used in the production of corn and chocolate syrup, production of low-calorie beer, purification of apple and pear juice, malt production, and in removing stickiness in the paper industry [2–4].

Microbial amylases have been generally favored over other plant’s and animal’s amylases because they are easily produced at low cost and in a shorter time. Moreover, the production of bacterial amylase is cheaper and faster than amylases produced by other microorganisms. Many industrial enzymatic reactions occurring at high temperatures have advantages in decreasing the contamination risk, increasing the diffusion rate, being resistant to denaturing agents, and proteolytic enzymes [5].

The amylases produced by different *Bacillus* species differ in their types, range of pH, and temperature [6]. The hot springs are a promising source for isolation of thermophilic bacteria producing thermo-stable α-amylase required in many industrial applications [7, 8]. The demand for thermostable amylases in biotechnology and industrial applications is increasing [9]. However, reports about bacterial strains that could produce thermostable amylase are still limited [10].

Statistical and mathematical techniques for predicting the behavior of process variables and explaining their interactions, and response surface methodology (RSM) have been applied in the optimization of amylase production [11–13].

## Methods

### Sampling and isolation of thermophilic bacteria

A soil sample from hot springs of Pharaoh’s bath (29° 12′ 24.9′′ N, 32° 57′ 35.4′′ E), Sinai, Egypt, was collected in a sterile container and kept in an icebox for isolation of thermophilic bacteria. The sample was 10-fold diluted in sterile 0.09% saline solution, then 100 μL from each dilution was cultured on nutrient agar plates. The plates were incubated at 70°C for 48 h. After the incubation period, bacterial colonies of distinctive morphology were selected and purified on fresh sterile nutrient agar plates.

### Selection of a degradative organism

The isolates were screened to produce amylase, cellulase, and caseinase through sub-culturing on agar plates supplemented with 1% soluble starch [14], carboxy methyl cellulose [15], and 15% skimmed milk [16], in triplicates, respectively. The plates were incubated at 70°C for 24 h. The isolate which produced the 3 hydrolytic enzymes was selected and identified.

### Molecular identification

Total DNA of the bacterial isolate was extracted according to the instruction manual using DNA extraction kits (Thermo, Fisher Scientifics, USA) and stored frozen at – 20°C until PCR reaction was carried out. A pair of flanking sequences, 16S-1F (5′-AGAGTTTGATCCTGGCTCAG-3′) and 16S-517R (5′-ATTACCGCGGCTGCTGG-3′), was used for primer binding sites to partially amplify the target gene. Two microliters of the bacterial DNA was used as a template for PCR reaction. PCR was carried on using Premix Taq (MyTaq, Bioline, UK) according to the instruction manual. PCR was performed in genius model FGENO2TD thermal cycler (Techne, England). The PCR conditions were adjusted to 5 min for initial denaturation at 94°C, then 35 cycles of 1 min at 94°C, 1 min at 54°C, and 1 min at 72°C, and finally 10 min at 72°C for amplification of genes. The amplified genes were run on 1% agarose gel with a size marker to determine the size and purity of the products. The amplified bands were cleaned using a PCR product purification kit (Thermo, Fisher Scientifics, USA).

Sequencing of forward directions of partial 16S rRNA gene was performed in Macrogen, Korea. The sequence was identified using the BLAST search program, National Center for Biotechnology Information (NCBI), and National Library of Medicine, USA [17].

Sequence alignments were performed by Clustal W 1.83 XP software, and a phylogenetic tree was constructed using the neighbour-joining method using MEGA 6 software. Then, the sequence was submitted using Bankit tool (NCBI website; www.ncbi.nlm.nih.gov) to obtain the accession number.

### Selection of the best amylase production medium

The selected isolate was inoculated on four different starch containing media, M1 medium of the following composition: 2% starch, 1% yeast extract, 0.1% peptone, 0.1% beef extract, 0.05% MgSO_4_, and 0.04% CaCl_2_ [18]; M2 medium: 0.5% soluble starch, 0.5% yeast extract, 0.25% (NH_4_)_2_SO_4_, 0.02% MgSO_4_·7H_2_O, 0.3% KH_2_PO_4_, and 0.025% CaCl_2_·2H_2_O [16]; M3 medium: 2% soluble starch, 0.3% yeast extract, 0.3% tryptone, 0.03% sodium dodecyl sulfate, 0.5% polyethylene glycol, 0.02% MgSO4 7H_2_O, 1.0% K_2_HPO_4_, and 1.0% NaCl (pH 7) [19]; and M4 medium :1% soluble starch, 0.2% yeast extract, 0.5% peptone, 0.05% MgSO_4_, 0.05% NaCl, and 0.015% CaCl_2_ (pH 7.0) [7]. Then, the flasks were incubated at 60°C for 24 h to determine highest activity.

### Detection of amylase activity

The amylase activity was assayed according to Miller [20] using 3,5-dinitrosalicylic acid (DNS) method through incubation of 300 μL of 1% starch in 50 mM sodium phosphate buffer (pH 7.5) with 200 μL cell-free supernatant for 30 min at 60, 70, and 80°C. The reaction was stopped by adding 500 μL DNS reagent and boiling it in a water bath for 10 min. After cooling at room temperature, the amount of glucose released was determined by measuring the absorbance at 550 nm using Unico 7200 Spectrophotometer. Amylase activity was estimated using a calibration curve for glucose over a concentration range of 15 to 100 μmol. One unit of enzyme activity was defined as the amount of enzyme that releases 1 μmol reducing sugars per min [21].

### Statistical factorial designs

The two experimental designs namely Full Factorial Design and Central Composite Design (CCD) were adopted to study the effects of four independent variables with their interactions on amylase activity and to determine the optimum variable level for maximum enzyme activity. The Minitab Software v. 18 program was used for designing the involved models and statistical-analyzing the obtained results of the response (α-amylase activity). Experiments were performed in 250 mL flasks containing 100 mL sterile M4 medium inoculated with 100 μL of (10^6^ CFU per mL) of the selected bacterial isolate. All experiments were triplicated and the mean amylase activity was calculated.

### Full factorial design

A 2^4^ full factorial design was used to study the statistical significance of the following variables: growth medium pH (A), incubation temperature (B), incubation period (C), and aeration (D) besides their interactions towards the amylase activity. Each factor was studied at two levels (low and high) chosen according to previous studies and literature review (Table 1). A total of 16 sets of experiments were carried out to determine the significant factors that affect the amylase activity.

**Table 1:** The investigated variables with their codes and levels for the full factorial design

Variables	Code	Low level (-1)	High level (+1)
pH	A	5	8
Temperature (°C)	B	50	75
Incubation period (h)	C	24	96
Aeration	D	Aerobic conditions	Anaerobic conditions

### Central composite design (CCD)

The central composite design is one of the designs in Response Surface Methodology (RSM). Variables that showed a significant effect in the previous full factorial model were inserted in a three-factor CCD to study their interactions and to determine the optimum variable level for maximum amylase production.

### Determination of amylase thermo-stability

The crude enzyme was incubated at different temperatures of 60, 70, 80, 90, and 100°C for zero, 30, 60, 90, and 120 min to determine the thermal stability using the DNS method. Thermal stability was expressed as percent residual activity, and the initial enzyme activity was taken as 100 % [22].

### Statistical analysis

The data obtained from both the full factorial and the RSM on amylase activity optimization were analyzed by analysis of variance (ANOVA) test. The regression of tested variables and their interactions, model significance, and the coefficient of determination (*R*^2^) of the generated models were estimated. The results were used to fit a polynomial model equation to represent the behavior of the system and correlate the relation between the studied variables and amylase activity. All experiments were triplicated, and the mean amylase activity was calculated.

## Results

### Isolation, screening, and identification of degradative bacterial isolate

The isolate growing on a nutrient agar plate, incubated at 70°C and the production ability of amylase, cellulase, and caseinase was selected.

**Fig. 1:**
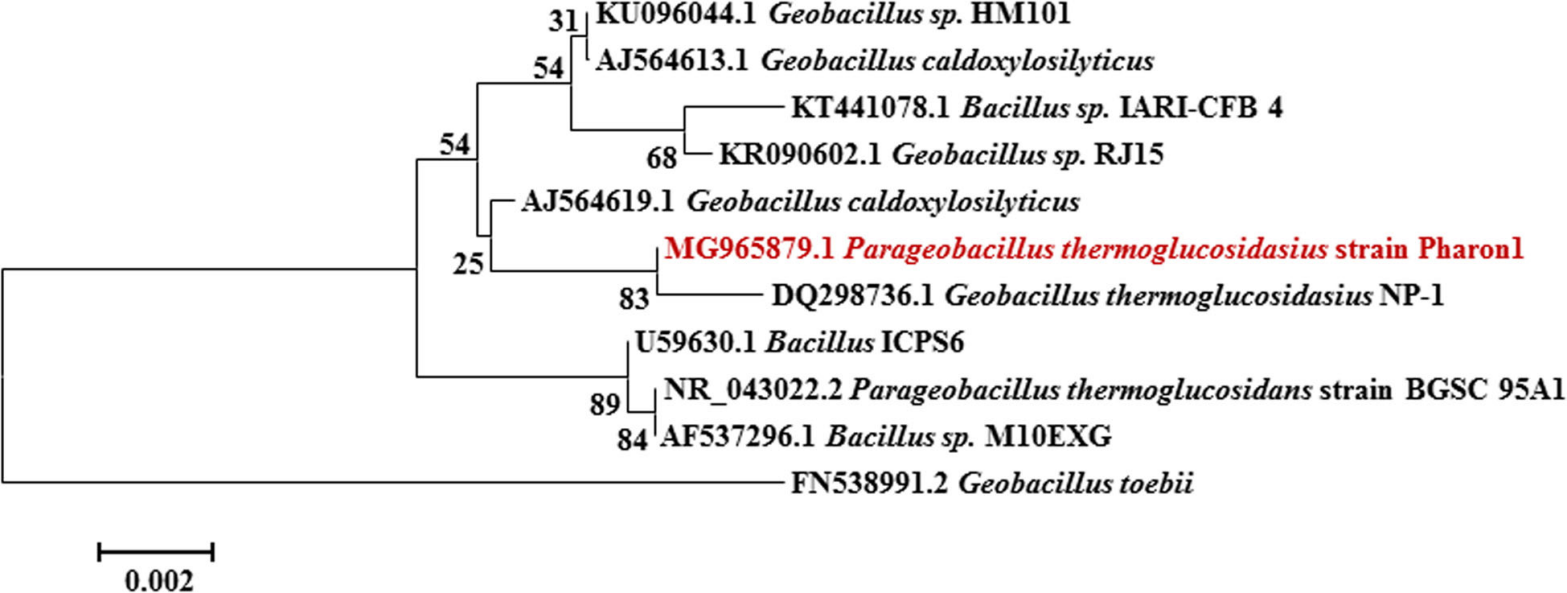
Neighbor joining phylogenetic tree of 16S rRNA genes. The numbers at the nodes are bootstrap values recovered from 100 trees, the bar indicates 0.2% nucleotide substitution

The isolate was identified by partial amplification and sequencing of 16S rRNA gene as *P. thermoglucosidasius* (99% sequence similarity). The nucleotide sequence was submitted to GenBank as *P. thermoglucosidasius* strain Pharon1 under accession number MG965879. Phylogenetic analysis with the alignment of the obtained gene sequence is illustrated in Fig. 1.

### Selection of the best production media

Medium containing 1% soluble starch, 0.2% yeast extract, 0.5% peptone, 0.05% MgSO_4_, 0.05% NaCl, and 0.015% CaCl_2_ (pH 7.0) [7] exhibited the highest amylase activity by *P. thermoglucogenesis* Pharaon1. Moreover, The DNS assay was carried out at different temperatures indicated that 70°C was the optimum temperature for the assay.

### Full factorial design

Table 2 presents the full factorial design matrix for the tested variables, given in uncoded values, plus the experimental and predicted data of amylase activity obtained by *P. thermoglucogenesis* Pharaon1. As shown, the highest enzyme activity was recorded in trial (12) when the bacterial strain was cultured at pH 5 and incubated aerobically at 50°C for 96 h.

**Table 2:** The full factorial design matrix showing the observed amylase activity of *P. thermoglucosidasius* Pharon1 exerted by the design trails

Run	Coded parameters				Mean amylase activity (U/mL/min)	
	***A***	***B***	***C***	***D***	Observed	Expected
1	8	50	96	Aerobic	0.0	0.3
2	5	50	96	Anaerobic	18.2	18.5
3	8	50	24	Anaerobic	0.0	0.3
4	5	75	96	Anaerobic	6.1	5.8
5	8	50	24	Aerobic	0.0	− 0.3
6	5	75	24	Anaerobic	2.1	2.4
7	8	75	96	Anaerobic	0.0	0.3
8	5	50	24	Anaerobic	5.0	4.7
9	8	75	96	Aerobic	0.0	− 0.3
10	5	75	96	Aerobic	17.4	17.7
11	5	50	24	Aerobic	32.0	32.3
12	8	75	24	Aerobic	0.0	0.3
13	8	75	24	Anaerobic	0.0	− 0.3
14	5	50	96	Aerobic	59.4	59.1
15	8	50	96	Anaerobic	0.0	− 0.3
16	5	75	24	Aerobic	3.3	3.0

*P. thermoglucosidasius* Pharon1 was inoculated in M4 medium [7] containing 1% soluble starch. Runs were triplicated, and the mean amylase activity was calculated

The ANOVA results in Table (S1) show that all of the tested factors of growth medium pH (A), incubation temperature (B), incubation period (C), and aeration (D) significantly (*P*<0.05) affect the amylase activity. Additionally, the 2-way and 3-way interacted terms of A*B, A*D, A*C, B*D, and A*B*D showed significant effects on the enzyme activity, while the interacted terms of B*C*D, B*C, A*B*C, C*D, and A*C*D showed non-significant effects on the same response under the same conditions.

Results also indicated that the regression model for this experimental design was highly significant (*P*<0.05). The coefficient of determination (*R*^2^) value was 0.9997 indicating a high correlation between the experimental and the predicted data at a confidence level of 95%.

The obtained results were fitted in a linear polynomial equation as the following:

$$ Enzyme\ activity=95.6-11.94\  pH-1.285\  Temp.+1.585\  Period-104.10\  Aeration+0.1607\  pH\ast Temp.-0.1981\  pH\ast Period+13.279\  pH\ast Aeration-0.01667\  Temp.\ast Period+1.4512\  Temp.\ast Aeration-0.2606\  Period\ast Aeration+0.002083\  pH\ast Temp.\ast Period-0.1857\  pH\ast Temp.\ast Aeration+0.02813\  pH\ast Period\ast Aeration+0.000569\  Temp.\ast Period\ast Aeration $$

### Response surface optimization

Factors of growth medium pH, temperature, and incubation period were represented in a CCD to determine their exact optimum levels for maximum amylase activity, while the aeration factor was categorical, and thus, it was kept at its optimum level, aerobic conditions as obtained from the tested full factorial model.

The design matrix of CCD is shown in Table 3. The results of amylase activity obtained in the CCD trials were evaluated through analysis of variance ANOVA results showed the significant effect (*P*<0.05) of each tested variable, A, B, and C in addition to all the squared variables, A^2^, B^2^, and C^2^ on the amylase activity by *P. thermoglucosidasius*. It was also noticed that all of 2-way interacted variables, A*B, A*C, and B*C showed a non-significant effect on amylase production. This model has a high *F* value and low *P* value (*P* < 0.05) that representing a good predictor for the responses (Table S2).

**Table 3:** Central composite design (CCD) matrix by the three tested variables besides the observed and expected amylase activity exerted by the model trials

Run	Coded parameters			Mean amylase activity (U/mL/min)	
	***A***	***B***	***C***	Observed	Expected
1	8.0	50.0	96.0	0	12.9
2	6.5	62.5	60.0	65	63.3
3	6.5	41.4	60.0	24.4	30.8
4	6.5	62.5	60.0	65.5	63.3
5	3.9	62.5	60.0	0	19.1
6	6.5	62.5	60.0	62.3	63.3
7	5.0	50.0	96.0	80.7	71.2
8	5.0	75.0	24.0	3.9	− 12.5
9	5.0	50.0	24.0	32.2	20.9
10	8.0	75.0	24.0	0	6.1
11	5.0	75.0	96.0	18.2	20.6
12	9.0	62.5	60.0	0	− 14.2
13	8.0	50.0	24.0	0	− 5.9
14	6.5	62.5	120.5	68.3	58.5
15	6.5	62.5	60.0	62.2	63.3
16	8.0	75.0	96.0	0	7.8
17	6.5	62.5	0.0	0	14.7
18	6.5	62.5	60.0	62.9	63.3
19	6.5	83.5	60.0	0	− 1.6
20	6.5	62.5	60.0	62.9	63.3

*P. thermoglucosidasius* Pharon1 was inoculated in M4 medium [7] containing 1% soluble starch under aerobic condition. Runs were triplicated, and the mean amylase activity was calculated

The coefficient of determination (*R*^2^) was 0.9081 reflecting 0.1% of the total differences in the responses could not be explained by this model. The quadratic model for predicting the optimal point was expressed according to a second-order polynomial equation as following:

$$ Enzyme\ activity=-574+88.7\  pH+9.63\  Temp.+2.775\  Period-9.57\  pH\ast pH-0.1101\  Temp.\ast Temp.-0.00729\  period\ast Period+0.605\  pH\ast Temp.-0.1454\  pH\ast Period-0.0095\  Temp.\ast Period $$

**Fig. 2:**
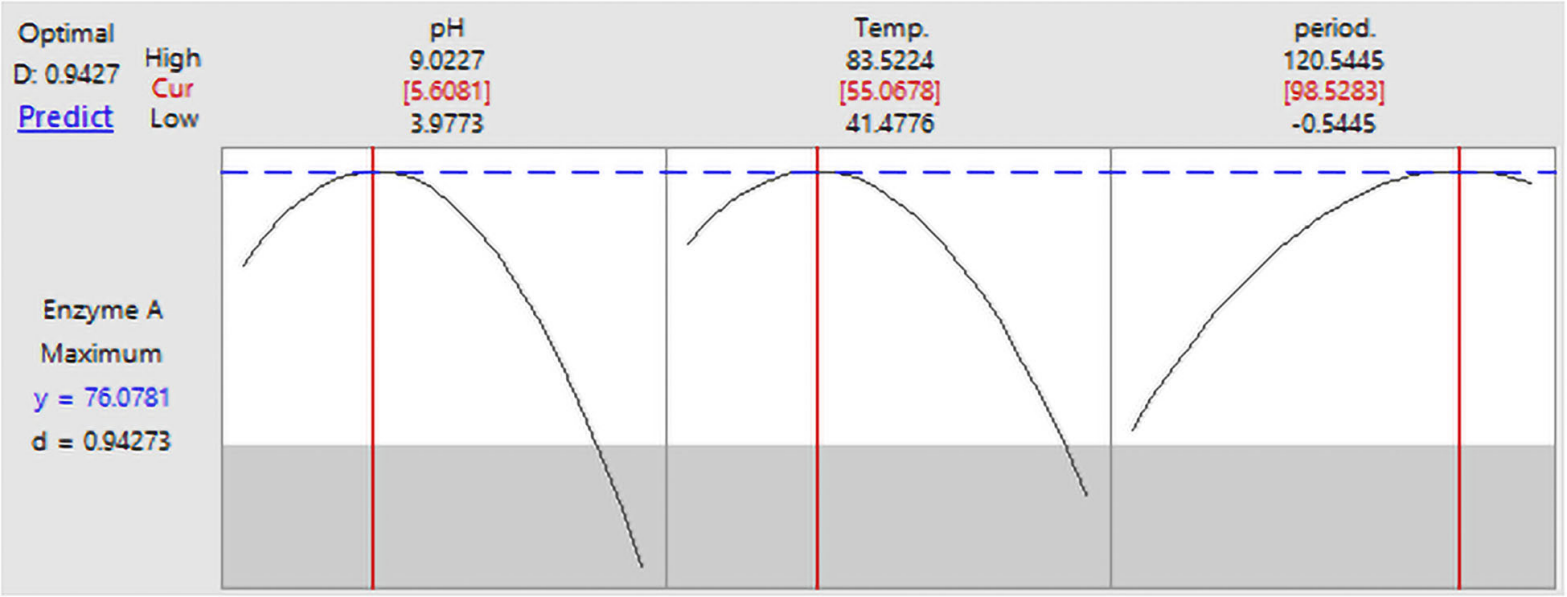
Predicted solution for the maximum amylase activity by *P. thermoglucosidasius* using the response optimizer of Minitab Software v. 18 defining the optimum levels of the selected three variables, growth medium pH, temperature, and incubation period. (Cur) is the curvature value (optimum), *y* is the amylase activity predicted, and *D* is the desirability value

**Fig. 3:**
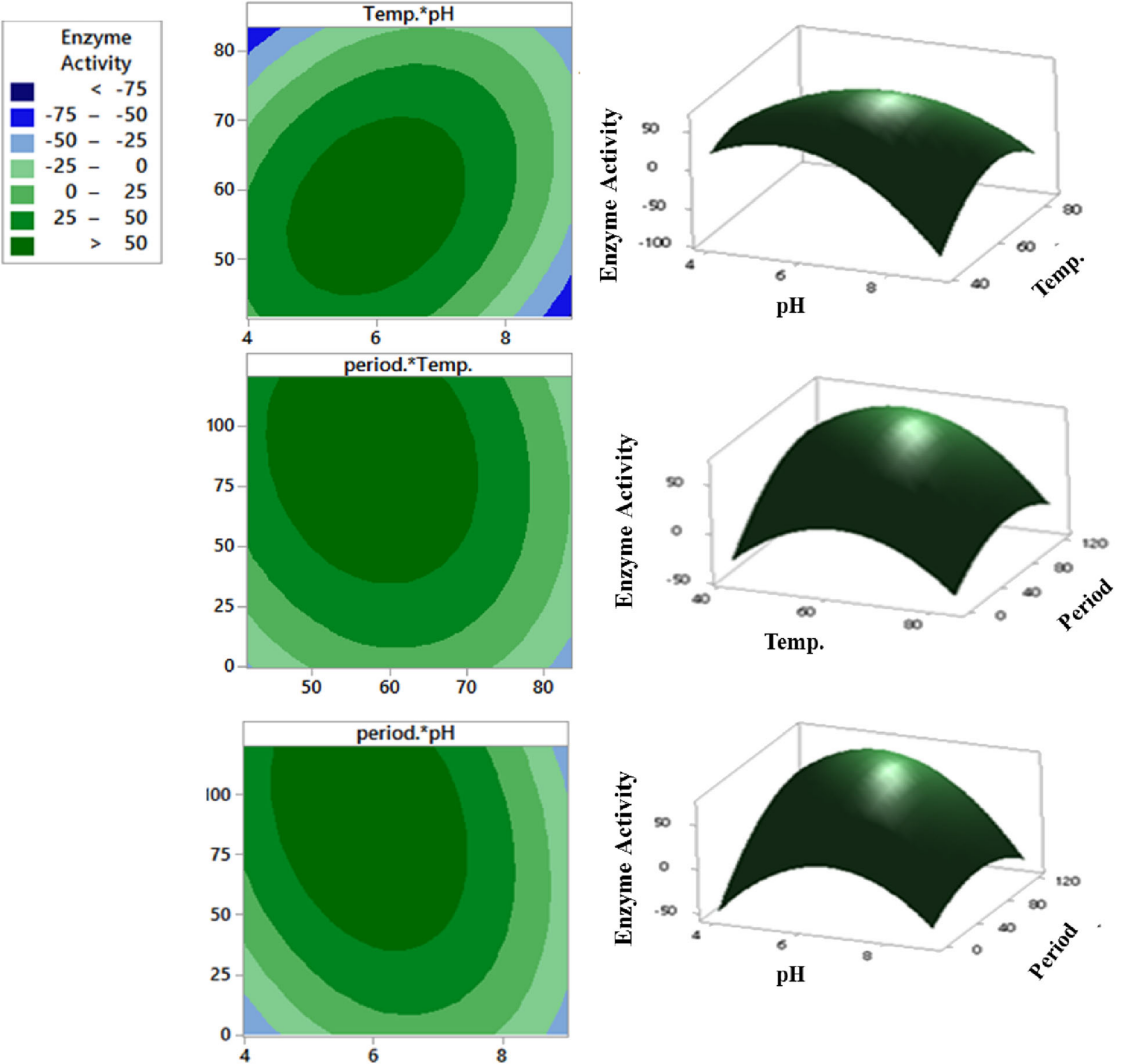
2D contour (on the left) and 3D surface (on the right) plots show the interaction effect and the optimum levels of the three significant factors on amylase activity

The maximum amylase activity achieved was 76.08 U/mL/min at pH 5.6, incubation temperature of 55°C for 98.5 h with a desirability of 94.27% as demonstrated by the response optimizer in Fig. 2. The 2D contour and 3D response surface plots showing the optimum level for each tested variable were represented in Fig. 3.

### Thermal stability

**Fig. 4:**
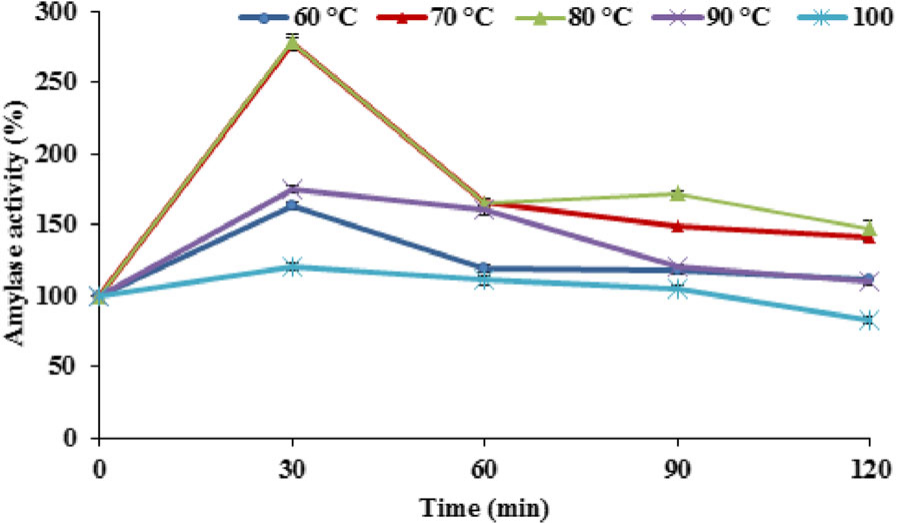
Thermo-stability of the produced amylase enzyme at different time intervals. Error bars represent SE (*n* = 3)

The thermo-stability of the crude enzyme was determined. It was found that the enzyme activity at room temperature was relatively low (100 %); but the highest activity (286 ± 4.65 %) was recorded after heating the enzyme at 70°C for 30 min, and the crude showed thermal stability as it was active even after exposure to 100°C for 120 min and lost about 13% from its activity (Fig. 4).

## Discussion

Thermophilic microorganisms with an optimum growth temperature of 50°C or above are sources of thermostable enzymes such as amylases, cellulases, chitinases, pectinases, xylanases, proteases, lipases, and DNA polymerases.

In this study, a thermophilic isolate was recovered from the hot springs region, Pharaoh’s Bath, Sinai, Egypt. Previous work by Saeed et al. [23] reported the isolation of thermophilic bacteria from the same site. Mohammad et al. [24] illustrated the isolation of closely related thermophiles from hot springs and deep sea in Jordon. Thermophiles exhibit maximum growth at 75°C [25]; therefore, the isolated bacteria recovered from this study are considered an extreme thermophile.

In this study, the isolate was screened for the production of amylase, cellulase, and caseinase production. The isolate showed its ability to produce the three enzymes which have significant biotechnological applications.

The selected isolate was identified by 16S rRNA gene sequencing as *P. thermoglucosidasius* strain Pharon1 (MG965879). *P. thermoglucosidasius* is a facultative anaerobic thermophilic bacterium which is frequently isolated from high-temperature environments including hot springs and compost [26]. The genus *Geobacillus* and its members have become potential for applications in biotechnology and bioremediation [27, 28] with a continually increasing industrial interest for their thermostable gene products [29, 30]. Thermophilic microorganisms with an optimum growth temperature of 50°C or above are sources of thermostable enzymes such as amylases, cellulases, chitinases, pectinases, xylanases, proteases, lipases, and DNA polymerases. These enzymes are appropriate for performing biotechnological processes at elevated temperatures [31].

Four different starch-containing media were inoculated with *P. thermoglucosidasius* strain Pharon1. After incubation for 24 h, the highest amylase activity was recorded using M4 medium as measured using the DNS assay method. The M4 medium was selected for the optimization of amylase production.

To optimize the physical conditions of the enzyme production, statistical approaches namely full factorial and central composite design were applied. Statistical optimization allows quick screening of large experimental domain and explains accurately the role of each tested variable and the combined effects of tested factors. The full factorial approach confirmed the significance of the four tested physical variables (pH, temperature, incubation period, and aeration) on the production of amylase enzyme. The obtained significant and numeric variables (pH, temperature, and incubation period) were used to design the central composite model which is an accurate tool in determining the optimum level for each factor with respect to the effect of the other factors [12, 32].

The maximum enzyme activity (80.6 U/mL/min) was accomplished when the bacterial strain was grown at a temperature at 54.1°C. This agreed with Arfah et al. [33] and Tiwari et al*.* [34] who reported that the optimum temperature of amylase production was at 55°C by *Bacillus* sp. RSII-1b and *Bacillus tequilensis* RG-01, respectively. However, other studies reported the production of amylases at a wider range of temperatures as mentioned and reported by Bekler et al. who obtained the maximum level of amylase from *Bacillus paralicheniformis* at 60°C [13].

Commercially, the interest has been focused on thermophilic amylase capable of functioning at a low pH range of 4.5–5.5 [35]. A similar result was obtained by Antrim et al. [36] who reported a thermostable α-amylase with activity at pH 5.5. Uguru et al. [37] isolated a strain of *Thermoactinomyces thalpophilus* which produced an extracellular amylase with optimum pH of 5.0. The thermostable amylases have extensive applications in a number of industrial processes [38].

The incubation of the crude enzyme preparation at 70°C for 30 min before adding the substrate resulted in 2.86 fold increase in its amylase activity compared to the enzyme preparation kept at room temperature, which means that the enzyme required thermal activation energy to exert its full activity. Thermostable amylase produced from *P. thermoglucosidasius* strain Pharon1 continued to be active over a temperature range. The thermostable enzyme produced in this study lost only 13% of its activity after 120 min of incubation at 100°C. Previous studies on *Bacillus subtilis* showed that 67% of the original activities were lost at 90°C [39].

It was also reported that α-amylases from *B. subtilis* KIBGE and *B. subtilis*, 65 showed optimal activities at 50°C and 60°C, respectively, and 28% reduction in enzyme activity was observed at 70°C, whereas incubation at 80°C resulted in complete inactivated of the enzyme [40].

Thermostable amylolytic enzymes are of great importance in modern biotechnological applications. They are used to produce many valuable products in different industries such as glucose, dextrose syrup, crystalline dextrose, maltose, and maltodextrins [41–43].

In conclusion, the enzyme produced by the novel local isolate, *P. thermoglucosidasius* strain Pharon1 (MG965879) showed strong degradative ability under extreme environmental conditions mark it as a potential candidate for biotechnological applications. Further characterization of the produced enzymes needs to be carried on.

## Conclusions

The novel bacterial strain *P. thermoglucosidasius* strain Pharon1 can be used in amylase production because it could be considered a cost-effective source for the enzyme. Where it requires a relatively low incubation temperature (55^o^C) to produce the thermophilic amylase at acidic pH of 5.6 within about 98 h of incubation; also, the enzyme produced showed thermostability after exposure to 100°C for 120 min.

## Supplementary Information

**Additional file 1: Table S1.** Statistical analysis for amylase activity in the full factorial design. **Table S2.** Statistical analysis in the central composite design.

## Data Availability

All data generated or analyzed during this study are included in this published article.

## Abbreviations

DNS: Dinitrosalicylic acid
CCD: Central composite design
RSM: Response surface methodology
*R*^2^: Coefficient of determination
