# Supplementary material for: Statistical factorial designs for optimum production of thermostable α-amylase by the degradative bacterium Parageobacillus thermoglucosidasius Pharon1 isolated from Sinai, Egypt
Source: J Genet Eng Biotechnol. 2021 Feb 1;19:24. doi: 10.1186/s43141-021-00123-4 (PMC7851196; doi:10.1186/s43141-021-00123-4)
Supplement: Supplementary file 1 — Additional file 1: Table S1. Statistical analysis for amylase activity in the full factorial design. Table S2. Statistical analysis in the central composite design. [file 43141_2021_123_MOESM1_ESM.docx]

**SUPPORTING INFORMATION**

Table (S1): Statistical analysis for amylase activity in the full factorial design

Table (S2): Statistical analysis in the central composite design

**Table (S1): Statistical analysis for amylase activity in the full factorial design**

| Source | DF | Adj SS | Adj MS | F-Value | P-Value |
| --- | --- | --- | --- | --- | --- |
| Model | 14 | 3975.80 | 283.99 | 270.30 | 0.048 |
| Linear | 4 | 2368.43 | 592.11 | 563.58 | 0.032 |
| pH | 1 | 1287.02 | 1287.02 | 1225.00 | 0.018 |
| Temp. | 1 | 459.03 | 459.03 | 436.91 | 0.030 |
| Period | 1 | 215.36 | 215.36 | 204.98 | 0.044 |
| Aeration | 1 | 407.03 | 407.03 | 387.42 | 0.032 |
| 2-Way Interactions | 6 | 1343.87 | 223.98 | 213.19 | 0.052 |
| pH*Temp. | 1 | 459.03 | 459.03 | 436.91 | 0.030 |
| pH*Period | 1 | 215.36 | 215.36 | 204.98 | 0.044 |
| pH*Aeration | 1 | 407.03 | 407.03 | 387.42 | 0.032 |
| Temp.*Period | 1 | 31.64 | 31.64 | 30.12 | 0.115 |
| Temp.*Aeration | 1 | 193.91 | 193.91 | 184.56 | 0.047 |
| Period*Aeration | 1 | 36.91 | 36.91 | 35.13 | 0.106 |
| 3-Way Interactions | 4 | 263.50 | 65.88 | 62.70 | 0.094 |
| pH*Temp.*Period | 1 | 31.64 | 31.64 | 30.12 | 0.115 |
| pH*Temp.*Aeration | 1 | 193.91 | 193.91 | 184.56 | 0.047 |
| pH*Period*Aeration | 1 | 36.91 | 36.91 | 35.13 | 0.106 |
| Temp.*Period*Aeration | 1 | 1.05 | 1.05 | 1.00 | 0.500 |
| Error | 1 | 1.05 | 1.05 |  |  |
| Total | 15 | 3976.85 |  |  |  |

DF, degree of freedom; Adj SS, Adjusted sum of squares; Adj MS, Adjusted mean of squares; F-value, Fisher’s value; P-value; indicates level of significance

The analysis of variance (ANOVA) was applied at 95 % confidence intervals. Variables and models would be statistically considerable at levels of significance, P value < 0.05.

**Table (S2): Statistical analysis and regression statistics in the central composite design**

| Source | DF | Adj SS | Adj MS | F-Value | P-Value |
| --- | --- | --- | --- | --- | --- |
| Model | 9 | 17090.9 | 1898.99 | 10.98 | 0.000 |
| Linear | 3 | 4918.5 | 1639.49 | 9.48 | 0.003 |
| pH | 1 | 1334.5 | 1334.49 | 7.72 | 0.020 |
| Temp. | 1 | 1272.7 | 1272.67 | 7.36 | 0.022 |
| period | 1 | 2311.3 | 2311.32 | 13.36 | 0.004 |
| Square | 3 | 10502.7 | 3500.88 | 20.24 | 0.000 |
| pH*pH | 1 | 6676.2 | 6676.19 | 38.60 | 0.000 |
| Temp.*Temp. | 1 | 4268.5 | 4268.47 | 24.68 | 0.001 |
| period.*period. | 1 | 1286.9 | 1286.87 | 7.44 | 0.021 |
| 2-Way Interaction | 3 | 1669.8 | 556.59 | 3.22 | 0.070 |
| pH*Temp. | 1 | 1030.6 | 1030.58 | 5.96 | 0.035 |
| pH*period. | 1 | 493.0 | 492.98 | 2.85 | 0.122 |
| Temp.*period. | 1 | 146.2 | 146.20 | 0.85 | 0.380 |
| Error | 10 | 1729.7 | 172.97 |  |  |
| Lack-of-Fit | 5 | 1719.6 | 343.92 | 170.37 | 0.000 |
| Pure Error | 5 | 10.1 | 2.02 |  |  |
| Total | 19 | 18820.6 |  |  |  |

DF, degree of freedom; Adj SS, Adjusted sum of squares; Adj MS, Adjusted mean of squares; F-value, Fisher’s value; P-value; indicates level of significance.

The analysis of variance (ANOVA) was applied at 95 % confidence intervals. Variables and models would be statistically considerable at levels of significance, P value < 0.05.
